# Altered brain metabolite concentration and delayed neurodevelopment in preterm neonates

**DOI:** 10.1038/s41390-021-01398-6

**Published:** 2021-03-05

**Authors:** Moyoko Tomiyasu, Jun Shibasaki, Hiroshi Kawaguchi, Mikako Enokizono, Katsuaki Toyoshima, Takayuki Obata, Noriko Aida

**Affiliations:** 1 Department of Molecular Imaging and Theranostics, National Institute for Quantum and Radiological Science and Technology, Chiba, Japan; 2 Department of Radiology, Kanagawa Children’s Medical Center, Yokohama, Japan; 3 Department of Neonatology, Kanagawa Children’s Medical Center, Yokohama, Japan; 4 Human Informatics Research Institute, National Institute of Advanced Industrial Science and Technology, Tsukuba, Japan; 5 Department of Radiology, Tokyo Metropolitan Children’s Medical Center, Tokyo, Japan

## Abstract

**Background:**

A very-low-birth-weight (VLBW) preterm infants is associated with an increased risk of impaired neurodevelopmental outcomes. In this study, we investigated how neonatal brain metabolite concentrations changed with postmenstrual age and examined the relationship between changes in concentration (slopes) and neurodevelopmental level at 3–4 years.

**Methods:**

We retrospectively examined 108 VLBW preterm infants who had brain single-voxel magnetic resonance spectroscopy at 34–42 weeks’ postmenstrual age. Neurodevelopment was assessed using a developmental test, and subjects were classified into four groups: developmental quotient <70, 70–84, 85–100, and >100. One-way analyses of covariance and multiple-comparison post hoc tests were used to compare slopes.

**Results:**

We observed correlations between postmenstrual age and the concentrations of *N*-acetylaspartate and *N*-acetylaspartylglutamate (tNAA) (*p* < 0.001); creatine and phosphocreatine (*p* < 0.001); glutamate and glutamine (*p* < 0.001); and myo-inositol (*p* = 0.049) in the deep gray matter; and tNAA (*p* < 0.001) in the centrum semiovale. A significant interaction was noted among the tNAA slopes of the four groups in the deep gray matter (*p* = 0.022), and we found a significant difference between the <70 and 85–100 groups (post hoc, *p* = 0.024).

**Conclusions:**

In VLBW preterm infants, the slopes of tNAA concentrations (adjusted for postmenstrual age) were associated with lower developmental quotients at 3–4 years.

**Impact:**

- In very-low-birth-weight preterm-born infants, a slower increase in tNAA brain concentration at term-equivalent age was associated with poorer developmental outcomes at 3–4 years.
- The increase in tNAA concentration in very-low-birth-weight infants was slower in poorer developmental outcomes, and changes in tNAA concentration appeared to be more critical than changes in tCho for predicting developmental delays.
- While tNAA/tCho ratios were previously used to examine the correlation with neurodevelopment at 1–2 years, we used brain metabolite concentrations.

## Introduction

A very low birth weight (VLBW) in preterm infants is associated with an increased risk of impaired neurodevelopmental outcomes, and early identification and intervention may effectively improve developmental outcomes.^1–6^ The incidence of major brain injuries has decreased with advances in neonatal care for preterm infants. However, some neonates who do not show significant brain injuries still exhibit later developmental disabilities.^4,7–9^ Thus comprehensive assessment of the prognosis of neonates must consider additional information.

Magnetic resonance spectroscopy (MRS) is a non-invasive technique for assessing cerebral metabolite levels. In predicting neurodevelopmental outcomes for LBW preterm infants, MRS has been used to measure the ratios of brain metabolites.^10–17^ However, studies have reported a number of different patterns, with some reporting correlations between the ratios of *N*-acetylaspartate and *N-*acetylaspartylglutamate (total *N*-acetylaspartate (tNAA)) to glycerophosphocholine (including choline-containing compounds) and phosphocholine (tCho), i.e., tNAA/tCho, and subsequent neurodevelopment.^11,13,15,16^ Since these ratios are affected by alterations in the levels of any of the above-mentioned metabolites, it is difficult to determine which metabolic disturbances cause neurological impairments, and slight pathological changes at the metabolite level may be overlooked. To address this in the present study, we retrospectively investigated neonatal brain metabolite concentrations in preterm-born infants with VLBW. Specifically, we examined whether the rates of change (slopes) in metabolite concentrations differed with postmenstrual age in subjects classified by neurodevelopmental level at 3–4 years. Regarding metabolite ratios, which are more clinically accessible values, we also investigated these changes with tCho as the denominator.

## Materials and methods

### Ethics statement

Our retrospective study was approved by the Institutional Ethical Review Board of our hospital, and the requirement to obtain written informed consent was waived.

### Subjects

**Fig. 1:**
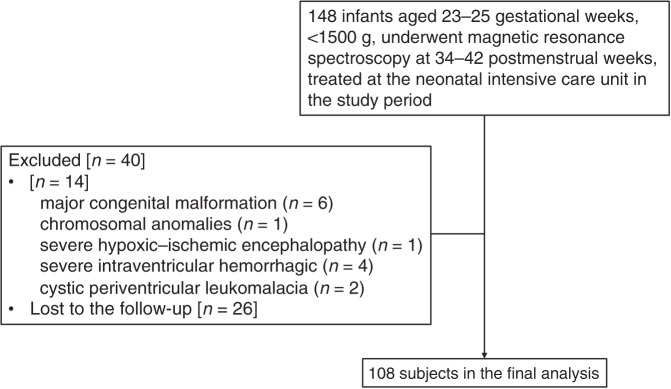
Flow chart of the patients included and excluded in the present study.

Our data were collected from a series of preterm patients admitted to the neonatal intensive care unit of our hospital between April 2013 and April 2016. The original cohort included 148 infants born at 23–35 gestational weeks with a birth weight of <1500 g, who were examined using brain proton magnetic resonance imaging (MRI) and MRS at a postmenstrual age of 34–42 weeks. Infants with major congenital malformations, chromosomal anomalies, severe hypoxic–ischemic encephalopathy, severe intraventricular hemorrhage, and/or cystic periventricular leukomalacia were not included in the dataset (*n* = 14). In addition, some infants did not undergo the follow-up neurodevelopmental examination at 3–4 years (*n* = 26). After these exclusions, data from 108 subjects were included in the present analysis (Fig. 1).

### Brain MRI and MRS examinations

**Fig. 2:**
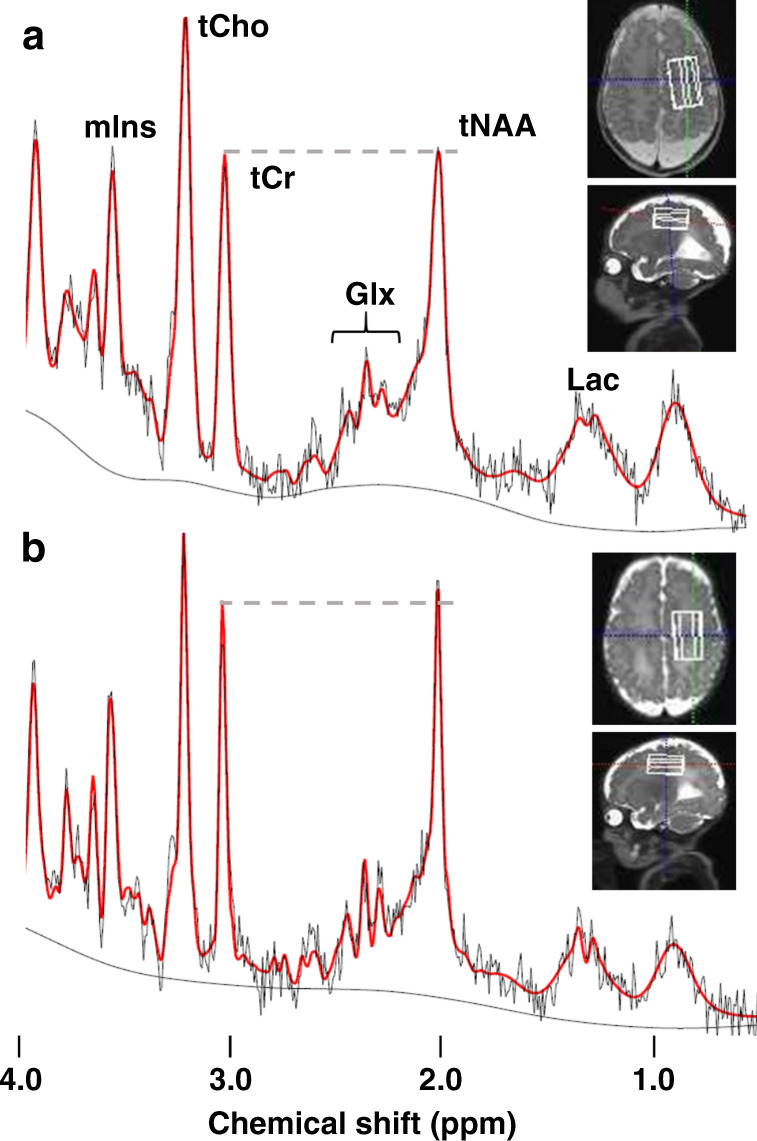
Representative LCModel outputs of in vivo proton magnetic resonance spectra for the centrum semiovale (volume, 5.6–5.8 mL; echo time, 30 ms; repetition time, 5,000 ms). **a** A neonate with a severely delayed outcome at a postmenstrual age of 40 weeks. **b** A neonate with a normal outcome at a postmenstrual age of 39 weeks. Thin lines show the original spectra, while bold lines represent the fitted lines produced by LCModel. The volumes of interest are overlaid on T2-weighted images. Glx glutamate and glutamine, Lac lactate, mIns myo-inositol, tCho glycerophosphocholine (including choline-containing compounds) and phosphocholine, tCr creatine and phosphocreatine, tNAA total *N-*acetylaspartate (*N-*acetylaspartate and *N*-acetylaspartylglutamate).

Each subject underwent one brain proton MRI and MRS examination during the neonatal period. All MRI and MRS scans were performed using a clinical 3-T magnetic resonance system (Magnetom Verio; Siemens Healthineers, Erlangen, Germany). Radio frequency signal transmission and reception were performed using a whole-body coil (bore diameter: 70 cm) and a 32-channel head coil (inner diameter: 22 cm), respectively. The excitation frequency was set to 2.38 ppm (123.20 MHz). During the MR examination, the neonates were placed in a horizontal supine position, wrapped in vacuum-type immobilization bags (CFI Medical Solutions, Fenton, MI), and heart rate and transcutaneous oxygen saturation were monitored continuously using a pulse oximeter (Nonin, Plymouth, MN). All MRS data included in the present study were collected as part of a routine brain MR examination conducted for clinical diagnostic purposes. Single-voxel MRS data were obtained using the point-resolved spectroscopic localization sequence^18^ with a water presaturation pulse. The echo time/repetition time (TE/TR) was 30/5000 ms, with 8–16 excitations. Volumes of interest (VOIs) occupied 2.0–7.6 mL and 2.4–7.4 mL volumes of the deep gray matter and the centrum semiovale, respectively (Fig. 2). The locations of VOIs were identified using transverse fast spin echo T2-weighted imaging (TE/TR, 119–123/5000 ms; field of view, 105–136 × 150 mm^2^; matrix size, 180–232 × 256; slice thickness, 2 mm; 44–56 slices without gaps) or 3D MP-RAGE T1-weighted imaging (TE/TR/inversion time, 2.14/1570/800 ms; field of view, 150 × 120 mm^2^; matrix size, 192 × 192; slice thickness, 1 mm). Spectra for the same VOIs were also obtained without the water presaturation pulse; these were used to correct eddy current-induced phase shifts and quantify metabolite concentrations. The total time required for the single-proton MRS examination of two VOIs was approximately 10 min.

### MR data quantification

MRS data processing, including signal quantification, was performed using the LCModel software (version 6.2–1G; http://s-provencher.com/lcmodel.shtml).^19^ Spectra with signal-to-noise ratios ≤4 were excluded from the present study. The signal-to-noise ratio, which is displayed in the LCModel output files, was defined as the ratio of the maximum spectral peak minus the baseline, divided by twice the root mean square residual.^19^ In the present study, the absolute concentrations of five major metabolites were measured: *N*-acetylaspartate and *N*-acetylaspartylglutamate (tNAA), creatine and phosphocreatine (tCr), glycerophosphocholine (including choline-containing compounds) and phosphocholine (tCho), glutamate and glutamine (Glx), and myo-inositol (mIns). To calculate the concentration of each metabolite, the water concentrations of the deep gray matter and centrum semiovale were set to 48.9 and 51.7 M, respectively,^20^ and the reduction of each metabolite and water peak according to the *T*_1_ and *T*_2_ values was also considered. *T*_1_ and *T*_2_ for the water in the deep gray matter and centrum semiovale were set to 1956 and 121 ms and 2566 and 206 ms, respectively. The *T*_1_ and *T*_*2*_ values of the metabolites were as follows^21,22^: the *T*_1_ values for tNAA, tCr, tCho, Glx, and mIns were 1310, 1660, 1180, 1180, and 1180 ms, respectively; the *T*_2_ values for tNAA, tCr, tCho, Glx, and mIns were 369, 199, 384, 199, and 199 ms, respectively. Supplemental 1 (online) includes details regarding MR spectroscopic signal quantification.

Metabolite ratios were obtained using the values displayed in the LCModel output files: tNAA/tCho, tCr/tCho, Glx/tCho, and mIns/tCho.

### Neurodevelopmental assessment

Neurodevelopmental outcomes were assessed using the Kyoto Scale of Psychological Development, a psychological developmental test, which is reported to closely correlate with the Bayley-III scale.^23^ The latest version of the Kyoto Scale of Psychological Development was standardized using data from 2677 Japanese children in 2001, and the developmental quotient (DQ) score was 100.6 ± 13.4 (mean ± 1 standard deviations (SD)).^24^ Psychologists who were blinded regarding the perinatal details of the subjects and the proton MRI and MRS data administered the test. A total of 102 children completed the test at 3 years of age, and 6 children who missed the 3-year follow-up appointment underwent the test at approximately 4 years of age. Thus 108 children (median age, 3.0 years; interquartile range, 3.0–3.1 years) provided follow-up data. Scores 1–2 SDs below standard norms were considered to indicate mild developmental delays, while moderate-to-severe developmental delays were indicated by scores of at least 2 SDs below the standard norms: (1) normal (DQ > 100); (2) borderline (DQ = 85–100); (3) mild delay (DQ = 70–84); and (4) moderate-to-severe delay (DQ < 70).^3^ Subjects with a Gross Motor Function Classification System (GMFCS) grade of 3–5, blindness without any fixation or following, profound hearing deficits, and/or epilepsy were defined as having a moderate-to-severe delay.

### Statistical analysis

Fisher’s exact test and Kruskal–Wallis test were used to compare the clinical features of the subjects. We used one-way analyses of covariance (ANCOVA) with postmenstrual age as a covariate and multiple-comparison post hoc tests to compare the rates of change (slopes) in metabolite concentrations and metabolite ratios with respect to age among the four groups classified by developmental outcomes.

All statistical analyses were performed using MATLAB (MathWorks, Natick, MA) or IBM SPSS Statistics 24 (IBM, Chicago, IL). *p* Values < 0.050 were considered to be significant.

## Results

The clinical characteristics of the subjects are shown in Table 1. Twelve out of 108 subjects (11%) were defined as normal, 44 (41%) as borderline, 32 (30%) as showing a mild delay, and the remaining 20 (19%) as showing a mild-to-severe delay (GMFCS score >2, one child; blindness, no children; profound hearing deficit, one child; epilepsy, no children). The child with a GMFCS score >2 and the child with deafness had DQ scores of 45 and 66, respectively. Significant differences were observed among the four developmental outcome groups in terms of sepsis (*p* = 0.01) and chronic lung disease (*p* = 0.02).

**Table 1:** Clinical features of the subjects (*n* = 108).

Developmental quotient score	>100	85–100	70–84	<70	*p* Value
	106 (103–110), *n* = 12	92 (85–100), *n* = 44	81 (70–84), *n* = 32	59 (45–69), *n* = 20	
Male sex^a^	3 (25)	18 (41)	18 (56)	12 (60)	0.15
Gestational age (weeks)	29.1 (24.9–33.6)	27.8 (23.0–34.3)	27.9 (23.9–35.4)	25.6 (23.3–35.9)	0.05
Birth weight (g)	933 (741–1434)	909 (518–1465)	892 (407–1473)	729 (473–1319)	0.01^b^
Postmenstrual age at MR examination (weeks)	36.3 (34.6–39.4)	36.9 (34.6–42.1)	37.3 (34.1–42.0)	38.0 (35.6–41.4)	0.06
Body weight at MR examination (g)	2027 (1486–2582)	2116 (1238–3280)	1980 (1346–2554)	1997 (1434–2910)	0.54
One-minute Apgar score	5.5 (2, 7)	6 (1, 8)	6 (2, 8)	5 (1, 8)	0.37
Five-minute Apgar score	7 (5, 9)	8 (4, 9)	8 (5, 9)	8 (6, 9)	0.84
Sepsis^a^	0 (0)	3 (7)	3 (9)	7 (35)	0.01^b^
Patent ductus arteriosus^a^	5 (42)	19 (43)	15 (47)	14 (70)	0.22
Necrotizing enterocolitis and/or localized intestinal perforation^a^	1 (8)	2 (5)	0 (0)	3 (15)	0.09
Chronic lung disease^a^	3 (25)	19 (43)	13 (41)	15 (75)	0.02^b^
Home oxygen therapy^a^	0 (0)	5 (11)	5 (16)	11 (55)	<0.01^b^

^a^number (percentage) or Data are presented as a median (range). Kruskal–Wallis test or Fisher’s exact test was used for statistical analyses.

^b^Significant difference (*p* < 0.05).

**Fig. 3:**
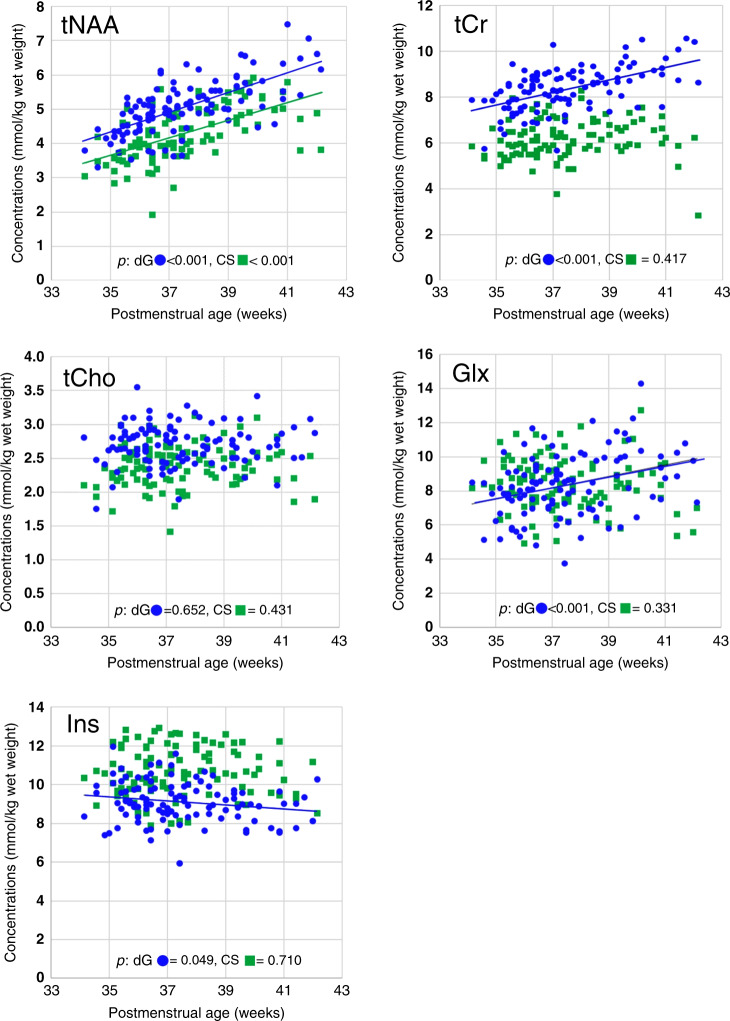
Scatterplots showing changes in metabolite concentration according to neonatal postmenstrual age (weeks) in the basal ganglia (*n* **=** 107, blue circle) and centrum semiovale (*n* **=** 105, green rectangle). Lines in the plot indicate the correlation between metabolite concentrations according to postmenstrual age, as calculated via a one-way analysis of covariance (*p* < 0.05). CS centrum semiovale, dGM deep gray matter, Glx glutamate and glutamine, mIns myo-inositol, tCho glycerophosphocholine (including choline-containing compounds) and phosphocholine, tCr creatine and phosphocreatine, tNAA total *N-*acetylaspartate (*N-*acetylaspartate and *N*-acetylaspartylglutamate).

In total, 212 MR spectra (107 deep gray matter and 105 centrum semiovale) were obtained from 108 subjects. MRS scans at the centrum semiovale level could not be performed in two patients due to a change in their clinical condition. In addition, two spectra, one each in the deep gray matter and centrum semiovale, were excluded from the study because they had a low signal-to-noise ratio of 4. The MR spectral signal-to-noise ratio was 12 (9–13) [median (interquartile range)], and the metabolite Cramér–Rao lower bounds, which are displayed as the percent SD in the LCModel fits, were as follows [median (interquartile range)]: tNAA, 7% (6–8%); tCr, 4% (4–5%); tCho, 4% (4–4%); Glx, 15% (13–17%); and mIns, 5% (5–6%). Among the overall metabolite concentrations, there were significant increases with postmenstrual age in tNAA (*p* < 0.001), tCr (*p* < 0.001), and Glx (*p* < 0.001) in the deep gray matter and tNAA (*p* < 0.001) in the centrum semiovale. In contrast, a significant decrease was observed with postmenstrual age in mIns in the deep gray matter (*p* = 0.049; Fig. 3). The metabolite ratios for which significant changes were observed are as follows: tNAA/tCho (*p* < 0.001), tCr/tCho (*p* < 0.001), Glx/tCho (*p* = 0.001), and mIns/tCho (*p* = 0.025) in the deep gray matter and tNAA/tCho (*p* < 0.001) in the centrum semiovale (Supplemental Fig. 1).

**Fig. 4:**
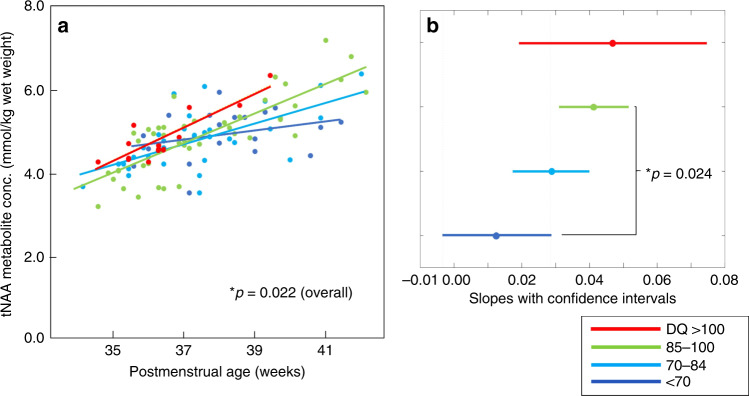
Changes in tNAA concentrations accoridng to postmenstrual age (weeks) in the deep gray matter from the four developmental groups. **a** Scatterplots of change in tNAA concentrations. **b** Slopes with comparison intervals from the data in **a**. Asterisk (*) represents significant difference (*p* < 0.050) revealed by: **a** a one-way analysis of covariance; and **b** post hoc multiple comparison. DQ developmental quotient, tNAA total *N*-acetylaspartate (*N*-acetylaspartate and *N*-acetylaspartylglutamate).

A significant interaction was observed among the tNAA slopes from the four developmental groups in the deep gray matter (postmenstrual age × group, *p* = 0.022 by ANCOVA; Fig. 4a), indicating that a slower rate of increase in tNAA concentrations was associated with a lower DQ score. Post hoc multiple comparisons revealed a significant difference in slopes from groups with DQ scores <70 and those with slopes from 85 to 100 (*p* = 0.024, Fig. 4b). While the slopes of the tNAA/tCho ratio in the deep gray matter approached significance for all four groups (*p* = 0.090), none of the slopes for the other metabolite concentrations or ratios showed significant interactions. Specifically, for the postmenstrual age × group interaction, the *p* values for tCr, tCho, Glx, and mIns in the deep gray matter were 0.647, 0.370, 0.595, and 0.378, respectively; those for tNAA, tCr, tCho, Glx, and mIns in the centrum semiovale were 0.847, 0.538, 0.969, 0.506, and 0.730, respectively; those for Glx/tCho, and mIns/tCho in the deep gray matter were 0.616, 0.831, and 0.844, respectively; and those for tNAA/tCho, Glx/tCho, and mIns/tCho in the centrum semiovale were 0.593, 0.178, 0.593, and 0.852, respectively.

## Discussion

In this retrospective study, we quantified the concentrations of brain metabolites in 108 preterm infants with VLBW who had no major brain injuries during the neonatal period. We found that overall changes in metabolite concentration were similar to those reported in previous studies.^25–28^ Our key results are as follows. First, children with lower DQ scores had slower rates of increase in tNAA concentrations in the deep gray matter (Fig. 4, *p* = 0.022). Second, compared with the tNAA/tCho ratio (*p* = 0.090), the rate of increase in tNAA concentration was more strongly correlated with neurodevelopmental status. Third, the metabolites included in the ratios that significantly changed with age (Supplemental Fig. 1) also changed in terms of concentration (Fig. 3). This indicates that metabolite ratios with tCho as the denominator may be most appropriate for evaluating changes in metabolite levels during the neonatal period, especially as it is difficult to obtain absolute metabolite concentrations at this time. Although previous studies have shown that the tNAA/tCho ratio is correlated with subsequent neurodevelopment,^11,13,15,16^ deducing which metabolites contribute significantly to changes in tNAA/tCho has been a difficult task. In the present study, changes in tNAA concentrations appeared to be more closely tied to developmental delays than changes in tCho concentrations.

Our finding regarding a relationship between the slope of the tNAA concentration (*p* = 0.022) and a lower developmental quotient is similar to recent findings reported by Chau et al., who found a relationship between slower increases in tNAA/tCho ratios in the basal nuclei and increasing severity of motor outcomes at a corrected age of 18 months (*p* = 0.02).^13^ However, we only observed a slight change in tNAA/tCho ratios (*p* = 0.090) in the present study. A possible explanation for this difference is that only one MRS examination was conducted per subject in the present study, and the sample size (*n* = 108) was smaller. In contrast, the study by Chau et al. included data from two MRS examinations per subject and had a larger sample population (*n* = 157). Therefore, repeated MRS examinations and a large sample size appear to be necessary for the detection of these differences. Furthermore, differences in the data acquisition methods (single-voxel MRS vs. multi-voxel MRS) and the age at neurodevelopmental assessment (3–4 years vs. corrected age of 18 months) may have influenced the obtained results.

Regarding the significant interaction found between the tNAA slope and DQ in the deep grey matter but not in the centrum semiovale, previous studies have also reported that both white matter and deep gray matter abnormalities were strongly associated with prognosis in preterm infants.^8,13,29–31^ For instance, Chau et al. reported a strong association between abnormal microstructure and metabolism in the basal nuclei, with adverse outcomes in preterm newborn.^13^ Further, Anderson et al. reported that structural abnormalities in the deep gray matter were particularly predictive of adverse neurodevelopmental outcomes.^31^ Regarding changes in the level of NAA, which is produced in neural mitochondria, a reduction in NAA is a well-known marker of neuronal or axonal loss in many neurological disorders. Further, the degree of NAA depletion is proportional to the degree of brain injury.^27,32–34^ Combining our data with previous findings, it is possible that changes due to microstructural damage may be more pronounced in deep gray matter compared with white matter, even if structural MRI reveals no changes. Alternatively, our selected VOI may have better reflected abnormalities in the deep gray matter. Indeed, as the white matter examined in the present study was from only one region, i.e., centrum semiovale, the MRS may not have captured abnormalities in the white matter region.

Our data showed that sepsis and chronic lung disease occurred more frequently in neonates with a poor developmental outcome at 3–4 years of age (Table 1). Previous studies have reported that neonatal sepsis and/or chronic lung disease could impair neurodevelopmental outcomes.^35,36^ Hüppi et al. demonstrated that developmental disruption and plasticity in the lungs and brain was closely associated with events inducing inflammation, oxidative stress, and endocrine disruption.^37^ These events could disturb NAA production in neuronal mitochondria,^32,33^ leading to reductions in tNAA concentrations in subjects with low DQ scores.

## Limitations

### Subjects

Although repeated MRS measurements are preferable to obtain rates of change (slopes) in metabolite concentrations, each subject in the present study underwent MRS examination only once during the neonatal period. Furthermore, the sample size (*n* = 108) was relatively small, particularly in the group defined as normal (DQ > 100, *n* = 12). This may be reflected in the slopes for this group, as the dataset had the broadest distribution (Fig. 4b). Further, neurodevelopment was assessed at 3–4 years of age, which is relatively young and may thus have suboptimal reliability for predicting neurodevelopment.^7^ A more long-term assessment, such as the Wechsler Intelligence Scale for Children (which is generally administered to children 6–16 years of age), may have yielded more detailed information regarding developmental deficits. Prospective studies with repeated MRS measurements, a larger sample size during the neonatal period, and neurodevelopmental evaluations at older ages are needed.

### Water concentration and relaxation times

Although we set the water concentrations as fixed values when estimating the metabolite concentrations, water concentrations in the brain are known to decrease with postmenstrual age, as are the *T*_1_ and *T*_2_ values for water.^20,38,39^ Williams et al. reported a strong correlation between R1 and R2, and a linear relationship between R1 and water concentration in the neonatal brain.^20^ This suggests that estimated metabolite concentrations at a younger postmenstrual age may need to be age-corrected. Taking this into account, the age-related changes in metabolite concentration (slopes) that we obtained may have been more moderate. However, after age correction, we still observed a significant difference in the tNAA in the deep gray matter among the four groups (ANCOVA, *p* = 0.042; post hoc, *p* = 0.046). Supplemental 1 (online) includes the detailed calculation method.

### Chemical shift displacements

In the PRESS sequence used in this study, the slice selection gradients led to chemical shift displacements of the metabolites and water peaks,^40^ i.e., each metabolite and water peak was spatially displaced against the excitation frequency (2.38 ppm). For a VOI of 20 × 20 × 20 mm^3^, tNAA peak displacement was approximately 0.3, 1.0, and 1.0 mm in the *x*, *y*, and *z* directions, respectively, while the water peak displacement was approximately 1.8, 6.2, and 6.2 mm in the *x*, *y*, and *z* directions, respectively. Given these displacements, cerebrospinal fluid outside of the VOI might have been included in infants with lower cerebral volumes.

## Conclusions

The slopes of the increases in tNAA concentrations in the deep gray matter in VLBW preterm infants during the neonatal period were associated with neurodevelopmental level at 3–4 years of age. The slopes of change in tNAA concentrations were more strongly related to developmental outcomes than those of tNAA/tCho ratios, suggesting that evaluations of separate changes in tNAA are preferable for predicting longer developmental delays. Further studies are needed to clarify the relationship between neonatal metabolite functions and neurodevelopmental outcomes in infants with VLBW.

## Supplementary Information

Supplemental Data

## Data Availability

Data are available from the corresponding author upon reasonable request.
