# Supplementary material for: Altered brain metabolite concentration and delayed neurodevelopment in preterm neonates
Source: Pediatr Res. 2021 Mar 5;91(1):197–203. doi: 10.1038/s41390-021-01398-6 (PMC8770132; doi:10.1038/s41390-021-01398-6)
Supplement: Supplementary file 1 — Supplemental Data [file 41390_2021_1398_MOESM1_ESM.docx]

***Supplemental Data***

***Magnetic Resonance Spectroscopy Signal Quantification***

To obtain the concentrations of N-acetylaspartate and N-acetylaspartylglutamate (total N-acetylaspartate; tNAA), creatine and phosphocreatine (tCr), glycerophosphocholine (including choline-containing compounds) and phosphocholine (tCho), glutamate and glutamine (Glx), and myo-inositol (mIns), the water concentrations of the deep gray matter and centrum semiovale were set at 48.9 and 51.7 M, respectively (20). The reduction of each metabolite and water peak according to the *T_1_* and *T_2_* values was also considered. We used the following formula:


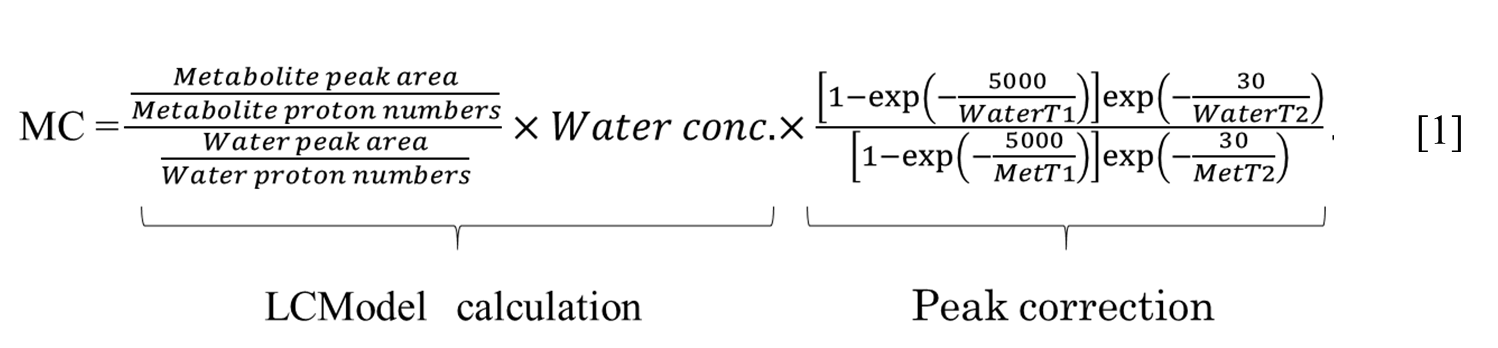


where MC is the metabolite concentration. The *T_1_* and *T_2_* values for water were calculated from the following equations, which were modified from previously published equations (20):

$T_{1}=\frac{{1000f}_{w}}{1.99-1.75f_{w}} (ms)$, [2]

$T_{2}=\frac{36T_{1}}{1000-0.215T_{1}} (ms)$, [3]

where *f_w_* is the water content, and T_1_ and T_2_ for the deep gray matter and centrum semiovale were calculated to be 1956 and 121 ms and 2566 and 206 ms, respectively.

The following values were used as the T_1_ and T_2_ values of metabolites based on published values from a 2.4 T study of the thalamus in term neonates (41): The T_1_ values for tNAA, tCr, and tCho were 1310, 1660, and 1180 ms, respectively; the T_2_ values for tNAA, tCr, and tCho were 369, 199, and 384 ms, respectively. The T_1_ and T_2_ values for mIns and Glx in neonates were set based on the lowest values among those for tNAA, tCr, and tCho (T_1_, 1180 ms; T_2_, 199 ms), because the T_1_ and T_2_ values for mIns and Glx in adults were found to be lower than those for tNAA, tCr, and tCho (21, 22).

Mlynárik *et al.* examined brain metabolites in adults and found the following: metabolite *T_1_* values at 3 T were close to those at 1.5, 2.0, and 4.1 T, whereas *T_2_* values decreased with increases in static magnetic field strength; metabolite *T_1_* values in the gray matter were higher than those in the white matter, while metabolite *T_2_* values were lower in the gray matter than those in the white matter (21). We estimated the differences in metabolite concentrations (calculated from Equation 1) that resulted from changes in *T_2_* expected to be caused by the strength of the static magnetic field were ≤ 6% lower at 2 T than at 3 T; with the value at 2.4 T positioned between these values. In terms of regional differences in metabolite *T_1_* and *T_2_* values, those in the centrum semiovale, which is a white matter region, were considered to be overestimates. However, differences in metabolite concentrations caused by regional differences were estimated to be within 5%.

**Correction of tNAA concentration using age-related changes in water T1, T2 and concentration**

For reference values, tNAA concentrations were corrected according to age-related changes in the T1 and T2 values of water and tNAA concentration (20): R1 = 1.99 x [1/water content] ‒ 1.75, R1 = 0.036 x R2 + 0.215, and R2 = 6.8 and 7.7, for 37 and 43 weeks postmenstrual age, respectively. R2 values were obtained from the basal ganglia graph in Fig. 3. We calculated that at the 37th postmenstrual week, the water concentration was 1.1% higher, T1 was 6% longer, and the T2 was 11% longer than at the 43rd postmenstrual week. Using Equation 1 shown above, we estimated the tNAA concentration at the 37th postmenstrual week to be 3% higher than that without the correction. Assuming that the rate of change in tNAA concentration due to the correction was linear with postmenstrual age, tNAA concentration changes with age (slopes) became more moderate. The result of a statistical analysis of the four slopes was as follows: ANCOVA, *p* = 0.042; post-hoc, *p* = 0.046.


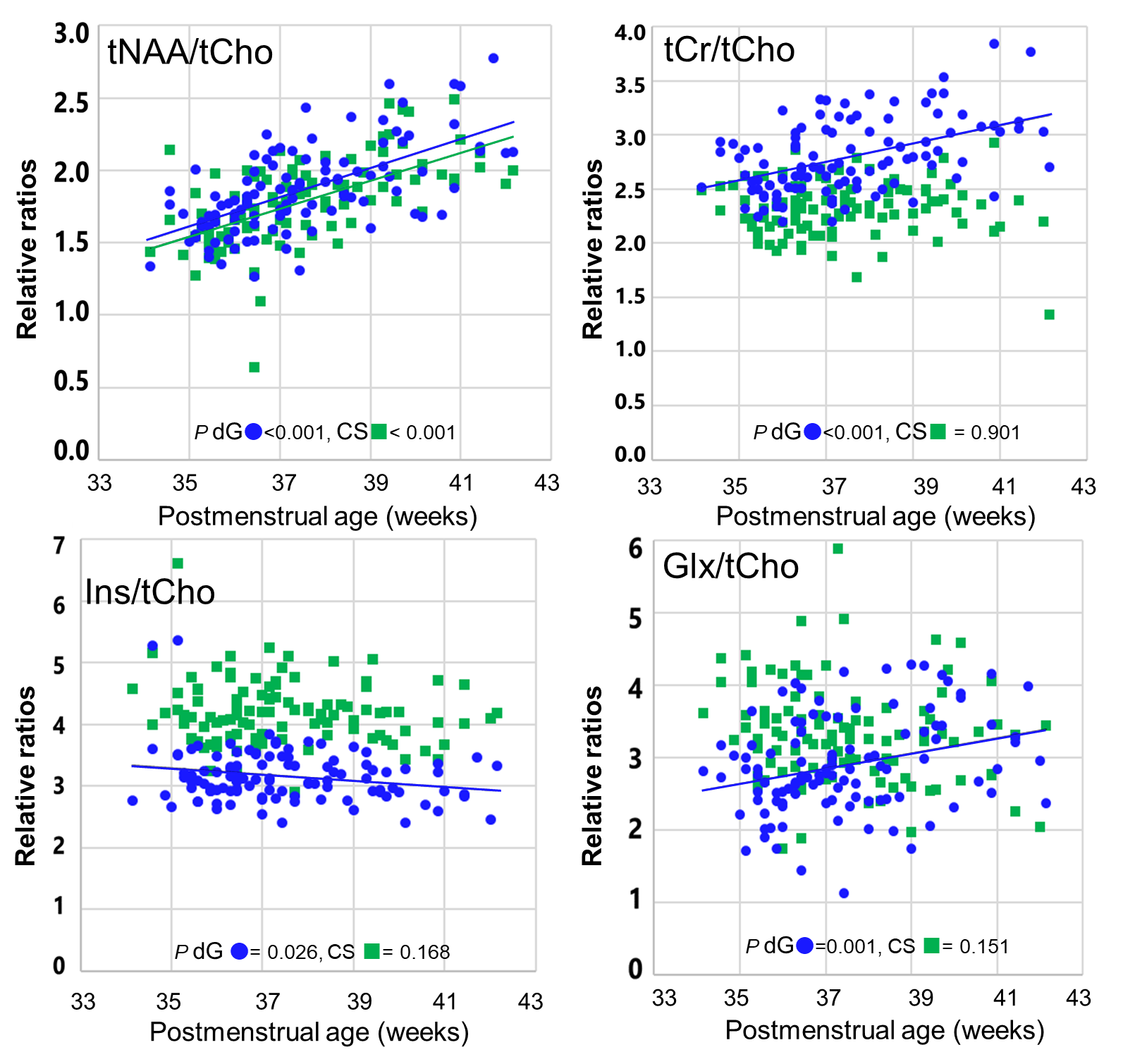


**Supplemental Figure 1. Scatterplots of change in metabolite ratios according to neonatal postmenstrual age (weeks) with tCho as the denominator. Blue circles represent the basal ganglia (*n* =107); green rectangles represent the centrum semiovale (n = 105).** Lines in the plot show the correlation between the concentration and postmenstrual age by a one-way analysis of covariance. Abbreviations: CS, centrum semiovale; dGM, deep gray matter; Glx, glutamate and glutamine; mIns, myo-inositol; tCho, glycerophosphocholine (including choline-containing compounds) and phosphocholine; tCr, creatine and phosphocreatine; tNAA, total N*-*acetylaspartate (N*-*acetylaspartate and N-acetylaspartylglutamate).

**
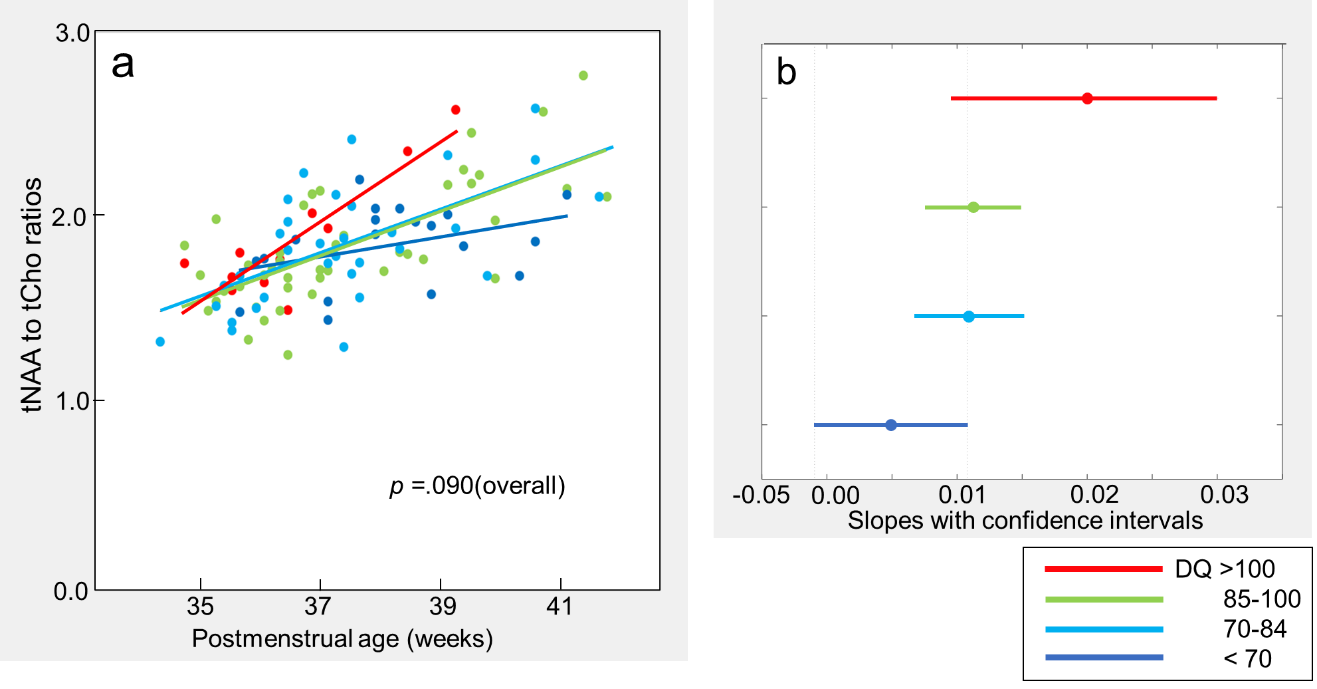
**

**Supplemental Figure 2.** a) Scatterplots showing change in the tNAA/tCho ratio in the deep gray matter according to postmenstrual age (weeks). A one-way analysis of covariance revealed no significant differences (*p* < 0.090). b) Lines represent the slopes of regression lines in (a). The subject groups were as follows. DQ > 100 (n = 12): red, DQ = 85–100 (n = 44): green, DQ =70–84 (n = 32): light blue, DQ < 70 (n = 20): blue. Abbreviations: tCho, glycerophosphocholine (including choline-containing compounds), and phosphocholine; tNAA, total N*-*acetylaspartate (N*-*acetylaspartate and N-acetylaspartylglutamate); DQ, developmental quotient.

**Reference**

1. Cheong JL, Cady EB, Penrice J, Wyatt JS, Cox IJ, Robertson NJ 2006 Proton MR spectroscopy in neonates with perinatal cerebral hypoxic-ischemic injury: metabolite peak-area ratios, relaxation times, and absolute concentrations. AJNR Am J Neuroradiol 27:1546-1554.
